# Critical thresholds of 1-Octen-3-ol shape inter-species *Aspergillus* interactions modulating the growth and secondary metabolism

**DOI:** 10.1038/s41598-020-68096-x

**Published:** 2020-07-06

**Authors:** Digar Singh, Su Young Son, Choong Hwan Lee

**Affiliations:** Department of Bioscience and Biotechnology, Konkuk University, Seoul, 05029 Korea

**Keywords:** Biological techniques, Analytical biochemistry, Mass spectrometry, Metabolomics, Microbiology techniques, Microbiology, Environmental microbiology, Fungi

## Abstract

In fungi, contactless interactions are mediated via the exchange of volatile organic compounds (VOCs). As these pair-wise interactions are fundamental to complex ecosystem, we examined the effects of inter-species VOCs trade-offs in *Aspergillus flavus* development. First, we exposed *A. flavus* to the *A. oryzae* volatilome (Treatment-1) with highest relative abundance of 1-Octen-3-ol (~ 4.53 folds) among the C-8 VOCs. Further, we examined the effects of gradient titers of 1-Octen-3-ol (Treatment-2: 100–400 ppm/day) in a range that elicits natural interactions. On 7-day, VOC-treated *A. flavus* displayed significantly reduced growth and sclerotial counts (*p* < 0.01) coupled with higher conidial density (T2_*100-200* *ppm/day*_, *p* < 0.01) and α-amylase secretion (T2_*200* *ppm/day*_, *p* < 0.01), compared to the untreated sets. Similar phenotypic trends except for α-amylases were evident for 9-day incubated *A. flavus* in T2. The corresponding metabolomics data displayed a clustered pattern of secondary metabolite profiles for VOC-treated *A. flavus* (PC1-18.03%; PC2-10.67%). Notably, a higher relative abundance of aflatoxin B1 with lower levels of most anthraquinones, indole-terpenoids, and oxylipins was evident in VOC-treated *A. flavus*. The observed correlations among the VOC-treatments, phenotypes, and altered metabolomes altogether suggest that the distant exposure to the gradient titers of 1-Octen-3-ol elicits an attenuated developmental response in *A. flavus* characterized by heightened virulence.

## Introduction

Volatile organic compounds (VOCs) are characterized by low molecular weight (50–300 Da) and high vapor pressure (≥ 0.01 kPa at 20 °C) which make them highly diffusible through local atmosphere, shaping a multitude of ecological interactions across the prokaryotic and eukaryotic species^1,2^. VOCs of microbial origin are ubiquitous in nature and are perhaps the most elusive of the biogenic chemical entities. Fungi produce a variety of VOCs which mediate their spatiotemporal interactions in an ecosystem. Approximately three hundred VOCs belonging to different chemical classes have been characterized in fungi; these include alcohols, aldehydes, ketones, aromatics, esters, heterocyclic hydrocarbons, monoterpenes, sesquiterpenes, and furans^3,4^. A few studies have suggested the antagonistic roles of fungal VOCs, mediating their cross-kingdom ecological interactions, whereby the growth and metabolism of interacting species are significantly modulated. Particularly considering the role of volatile oxylipins in fungal interactions, C-8 (eight C-atoms) fungal VOCs including 1-Octen-3-ol, have been reported to inhibit the growth of pathogenic fungi, including *Botrytis cinerea* and *Lecanicillium fungicola* that infect plants and commercial mushrooms, respectively^5,6^. Additionally, the fungal volatilome can also modulate the tri-trophic interactions between fungi, plants, and insects, making the host plants more susceptible to arthropod herbivory^7^. In di-trophic fungal-insect interaction, the C-8 fungal VOCs are thought to act as chemical signaling molecules that elicit behavioral responses in insects to aid the establishment of symbiotic relationships or escape from the fungivory, thereby increasing their competitive survival rates^8,9^.

Considering the infochemical function of VOCs for shaping the inter-/intra-species fungal interactions, C-8 VOCs have been reported to inhibit conidia germination and mycelial growth in Ascomycetes, through reversibly inducing microcycle conidiation with altered membrane permeability and intracellular pH^10–12^. Previously, we have reported the VOCs mediated intra-species interactions between *A. oryzae* strains used as inocula (trivially: *nuruk)* in artisanal soy food fermentation^13,14^. We observed that the C-8 VOCs (1-Octen-3-ol, 3-Octanone, and 2-Octenal) were the most abundant and likely influenced the growth, sclerotia development, enzymes secretion, and the metabolite profiles among the interacting *A. oryzae* strains. Notably, the sclerotia development also serves as marked phenotype in some toxicogenic strains of *A. flavus*, where this morphological transformation and secondary metabolism, including aflatoxin biosynthesis are closely linked through global regulator genes like *laeA* and *veA*^15^. *A. flavus* has been reported to exhibit reduced aflatoxin production, coupled with decreased sclerotial formation, in response to exogenously induced oxidative stress, which prevents its timely morpho-transformation^16^. Furthermore, reduced mycelial growth, coupled with enhanced conidiation and aflatoxin biosynthesis in response to abiotic stress factors, such as visible light has been reported for *A. flavus*^17^.

Though 1-Octen-3-ol is ubiquitous and its importance in the context of the growth and development of *Aspergillus* species is well-recognized, the mechanisms linking its semiochemical function to its specific effects on secondary metabolism in fungi, are largely unknown. In this study, we explored the effects of 1-Octen-3-ol gradients on growth, conidiation, morpho-transformation, enzyme secretion, secondary metabolism, and the overall virulence of *A. flavus*. We also aimed to estimate the likely interactions of *Aspergillus* in specific fermented food matrices, where the C-8 VOCs are produced and shared among inoculated molds, for example, between *A. oryzae* and toxicogenic contaminant species such as *A. flavus.*

## Results

### C-8 VOCs in *A. oryzae* headspace volatilome influence *A. flavus* development

**Figure 1:**
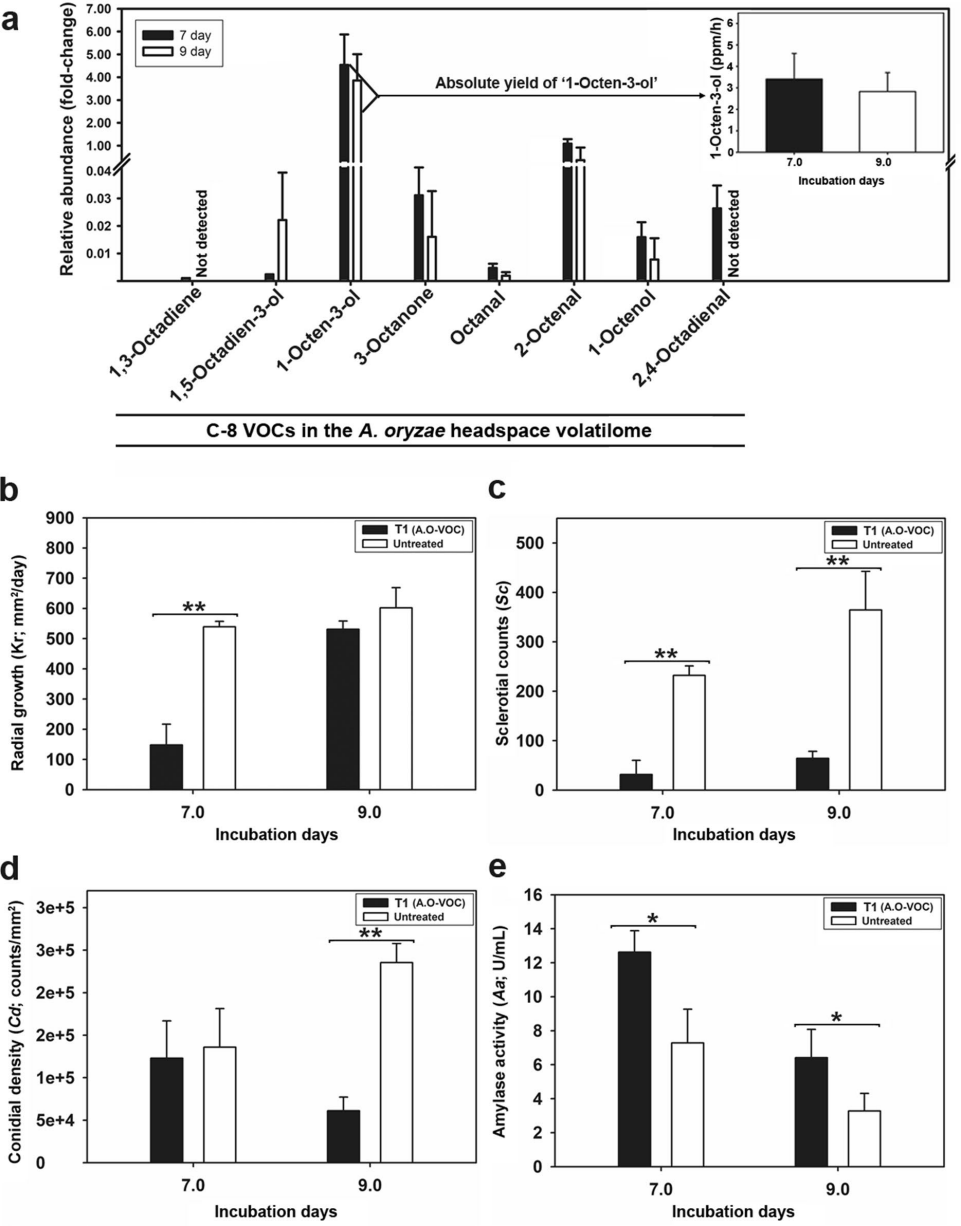
(**a**) The time-correlated (7 and 9 days) relative abundance of C-8 VOCs and the absolute production yield of 1-Octen-3-ol (inset) in the headspace volatilome of *A. oryzae* incubated in plate 2 (P2: source of VOCs) of twin-plate. Effects of treatment 1 (T1—*A. oryzae* volatilome) on the developmental phenotypes (**b**) radial growth rates, *Kr* (**c**) sclerotial counts, *Sc* (**d**) conidial density, *Cd* and (**e**) the secreted α-amylase activity, *Aa* of *A. flavus* incubated in plate 1 (P1: sink of VOCs) during the twin-plate experiment. The statistical significance between the phenotype observations were evaluated using one-way ANOVA with Duncan's multiple range tests at *p < 0.05 and **p < 0.01. The data represents the mean value for three biological replicates with error bars indicating the standard deviation.

Previously, we have reported that C-8 compounds were the most abundant VOCs in the headspace of *A. oryzae*^13^. Herein, we first examined the production profiles of C-8 VOCs in the *A. oryzae* headspace during its twin-plate incubation (7–9 days) with partner strain *A. flavus* (plate 1). Overall, we identified eight different C-8 VOCs namely 1,3-Octadiene; 1,5-Octadien-3-ol; 1-Octen-3-ol; 3-Octanone; Octanal; 2-Octenal; 1-Octanol; and 2,4-Octadienal. The VOCs were characterized by matching their chromatographic characteristics (retention time, RT) and the pattern of mass fragment ions with available databases, *in house* library, and the standard compounds used in the present work. Most notably, 1-Octen-3-ol displayed the highest relative abundance and quantified as 4.53 folds on 7-day and 3.86 folds on 9-day of incubation compared to internal standard (Fig. 1a). Pertaining to the significantly higher abundance and associated info-chemical functions of 1-Octen-3-ol, we focused on determining its absolute yield during the in vitro *Aspergillus* interactions in twin-plate assembly. The targeted quantitative analyses of *A. oryzae* headspace volatilome indicated that the freshly harvested culture produced approximately 3.40 ± 1.19 ppm/h of 1-Octen-3-ol on 7-day of incubation. Since the observed yield of the VOC was recorded for the mycelial biomass following the 1 h post-harvest incubation, we extrapolated the 1-day yield to approximately 82 ppm/day. However, the 1-Octen-3-ol production rate was reduced to 2.82 ± 0.88 ppm/h on 9-day indicating an intra-day yield of 68 ppm/day (inset of Fig. 1a).

Further, we examined the effects of *A. oryzae* volatilome with the highest relative abundance of 1-Octen-3-ol (treatment 1; T1) on the growth and developmental phenotypes of *A. flavus* incubated in plate 1 (P1) of twin-plate assembly. On 7-day of incubation, we recorded the significantly lower radial growth rate (*K*_*r*_) and sclerotial count *(Sc)* coupled with marginally lower conidial density *(Cd)* and the significantly higher secretory amylase activity *(Aa)* for *A. flavus* subjected to T1, compared to the untreated sets at *p* < 0.01 (Fig. 1b–e). However, the 9-day incubated *A. flavus* displayed marginally lower *K*_*r*_ with significantly lower *Sc* and *Cd* coupled with significantly higher *Aa* compared to untreated sets at *p* < 0.01. Taken together, these results indicate that 1-Octen-3-ol which constitutes the largest proportion of C-8 VOCs in the *A. oryzae* volatilome may have affected *A. flavus* development while its twin-plate incubation.

### Exposure to the gradient titers of 1-Octen-3-ol influence *A. flavus* development

We assumed that the temporal production dynamics of 1-Octen-3-ol must be higher than the calculated yield owing to the probable random errors that may have arisen during the multi-step process involving sample harvest from twin-plate, biomass transfer into SPME vial, altered physicochemical conditions during post-harvest incubation in Ringer's solution, VOC extraction, and instrument analysis^18^. Hence, we considered the higher concentration gradients (> 100 ppm) of standard 1-Octen-3-ol toward examining its potential effects on *A. flavus* developmental phenotypes (growth, sclerotia morphogenesis, conidiation, and amylase production).

**Figure 2:**
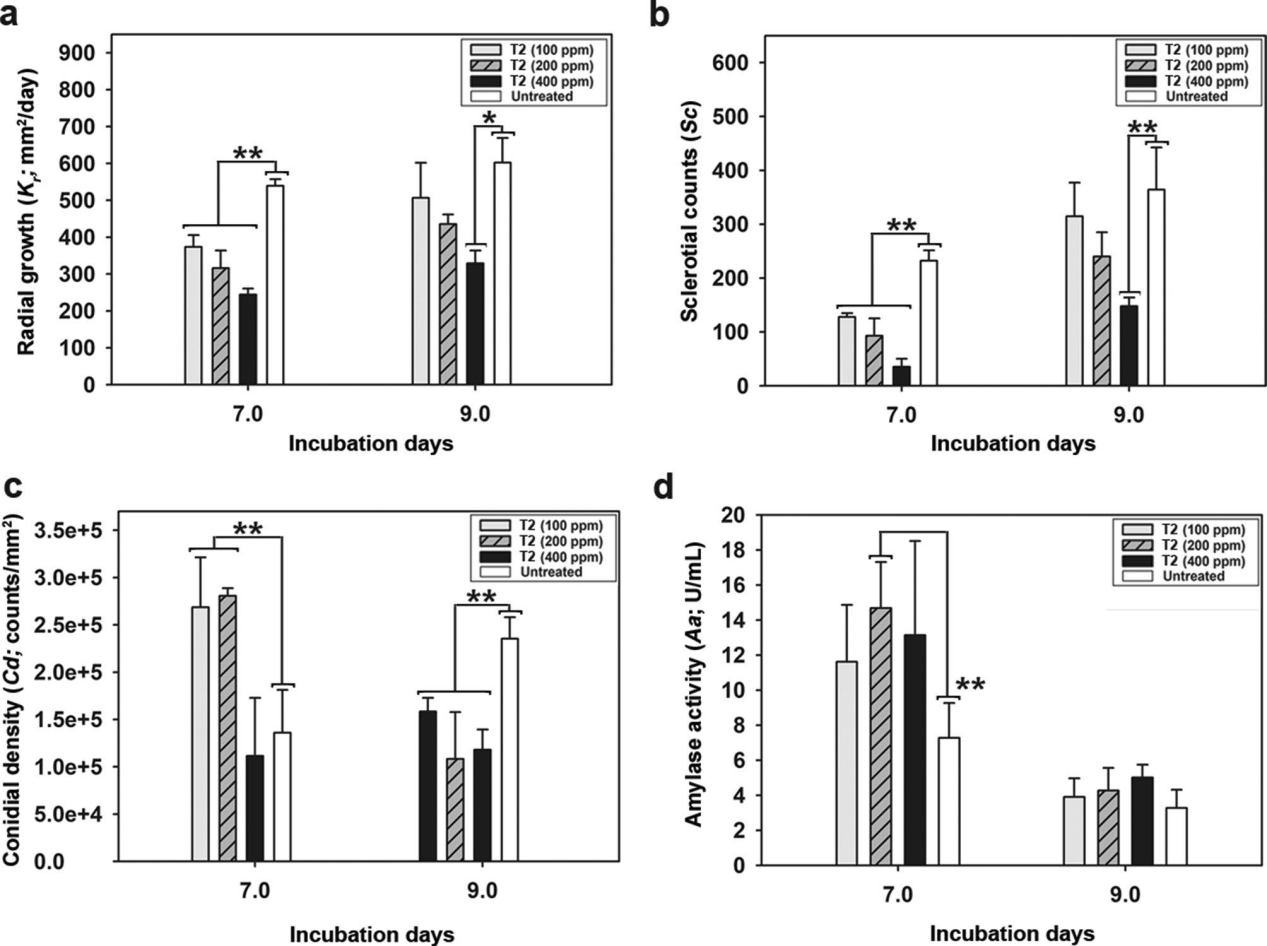
Effects of treatment 2 (T2—1-Octen-3-ol gradients) on the developmental phenotypes (**a**) radial growth rates, *Kr* (**b**) sclerotial counts, *Sc* (**c**) conidial density, *Cd* and (**d**) the secreted α-amylase activity, *Aa* of *A. flavus* incubated in plate 1 (P1: sink of VOCs) during the twin-plate experiment. The data represent the mean for three biological replicates with error bars indicating the standard deviation. The statistical significance between the observations were evaluated using one-way ANOVA with Duncan's multiple range tests at *p < 0.05 and **p < 0.01.

Most notably, we observed an inverse correlation between the 1-Octen-3-ol gradient treatment (T2) and *A. flavus* growth. The twin-plate exposure of *A. flavus* to the increasing titers of 1-Octen-3-ol resulted in the following order of growth rates (*K*_*r*_); untreated > T2 _*100* *ppm/day*_ > T2 _*200* *ppm/day*_ > T2 _*400* *ppm/day*_ on 7-day of incubation (*p* < 0.01). Similar trends were evident for the *K*_*r*_ values from 9-day incubated *A. flavus* except for a time-correlated increase in overall growth rates (Fig. 2a). Considering the morpho-transformation as an important stage in *A. flavus* growth cycle, we investigated the effects of 1-Octen-3-ol gradient exposure on sclerotia formation. Intriguingly, we observed the reciprocal trends for the total sclerotial counts *(Sc)* in *A. flavus* subjected to the increasing concentration gradients of 1-Octen-3-ol. The *Sc* varied significantly between the treated (T2) and untreated samples (*p* < 0.01), with the following trend; untreated > T2 _*100* *ppm/day*_) > *Sc* T2 _*200* *ppm/day*_ > *Sc* T2 _*400* *ppm/day*_, on 7 and 9-day of incubation (Fig. 2b).

The standard VOC treatments varyingly influenced the conidia formation in *A. flavus* depending upon the different titers of 1-Octen-3-ol treatments (Fig. 2c). On day 7 of incubation, *A. flavus* exposed to lower titers (T2 _*100–200* *ppm/day*_) of standard 1-Octen-3-ol displayed significantly higher conidial density (*Cd*) compared to the corresponding treatment with its higher titers (T2 _*400* *ppm/day*_) as well as the untreated sets, (*p* < 0.01). Intriguingly, we observed a critical threshold of 1-Octen-3-ol treatment where its varying concentrations, i.e., ≤ 200 ppm/day and 400 ppm/day resulted higher and lower *Cd*, respectively. However, the *Cd* values for 9-day incubated treated sets (T2 _*100–400* *ppm/day*_) were significantly lower compared to the untreated sets (*p* < 0.01).

Secretory α-amylase production is considered as the function of *A. flavus* ability to colonize host surfaces (especially the plants) and its virulence^19^. Notably, the higher α-amylase activities (*Aa*) were observed for *A. flavus* following the 1-Octen-3-ol gradient treatments in the following order; T2 _*200* *ppm/day*_ > T2 _*400* *ppm/day*_ > T2 _*100* *ppm/day*_ > untreated, on 7-day of incubation (Fig. 2d). However, the α-amylase production was considerably decreased in all the treated as well as control sets on 9-day of incubation. These observations are consistent with our earlier report suggesting the higher extracellular secretion of hydrolytic enzymes in *Aspergillus* species following the VOCs exposure, including the C-8 alcohols^13^.

### Exposure to 1-Octen-3-ol influence secondary metabolite profiles in *A. flavus*

**Figure 3:**
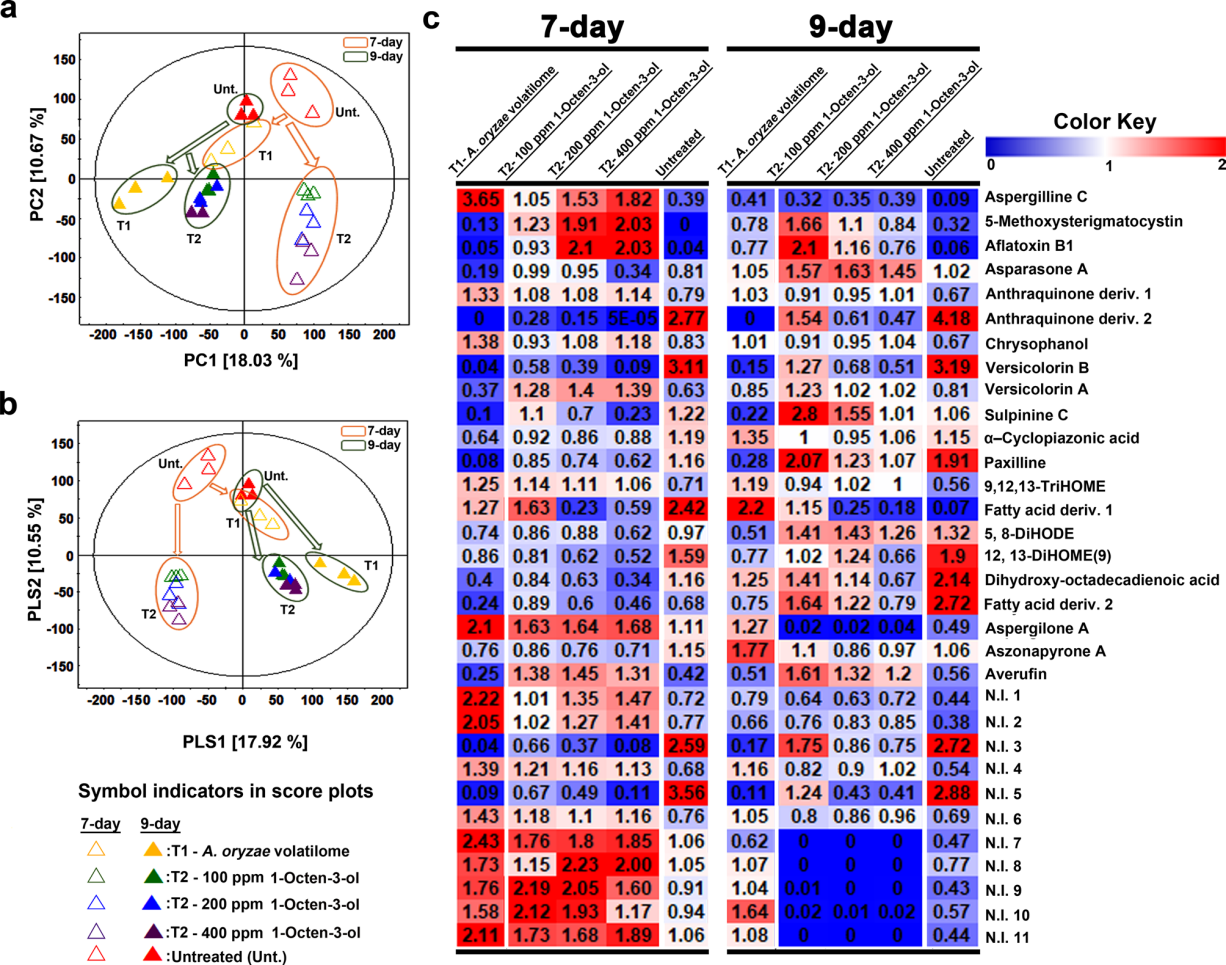
The multivariate score plots (**a**) PCA, (**b**) PLS-DA and (**c**) the heat-map for the relative abundance of significantly discriminant metabolites (VIP > 0.7 and *p* < 0.05), based on the UHPLC-LTQ-Orbitrap-MS datasets. The data highlights a marked disparity in the secondary metabolite profiles of *A. flavus* subjected to varying VOC treatments (T1 and T2) compared to the untreated sets, on 7 and 9 days of incubation during twin-plate experiment.

Having established that 1-Octen-3-ol affects the normal course of growth and development in sclerotia forming strain of *A. flavus*, we examined its effects on associated secondary metabolite profiles. The multivariate analyses based on UHPLC-LTQ-Orbitrap-MS datasets displayed considerable disparity in the secondary metabolite profiles of *A. flavus* exposed to *A. oryzae* volatilome (T1) as well as the varying gradients of 1-Octen-3-ol (T2) in the twin plate assembly. The unsupervised principal component analysis (PCA) score plot indicated clearly distinct patterns of secondary metabolite profiles for *A. flavus* subjected to different VOC treatments. Notably, the datasets for 7-day incubated *A. flavus* exposed to *A. oryzae* volatilome (T1) as well as the untreated sets displayed clustered profiles segregated across PC2 (10.67%) from those subjected to 1-Octen-3-ol gradient treatments (T2). Further, the metabolite profile datasets for *A. flavus* (7-day) subjected to T1 were segregated across PC1 (18.03%) from those observed for treatment sets T2 and the untreated (Fig. 3a). However, the metabolite profiles for 9-day incubated *A. flavus* showed only two major dataset clusters, with all treatments sets (T1 and T2) clustered and segregated from the untreated groups across PC1, suggesting a time correlated and transient effect of 1-Octen-3-ol exposure (Fig. 3a).

Similar to PCA, the supervised partial least squares—discriminant analysis (PLS-DA) indicated a marked variability in the metabolite profiles of VOC treated (T1 and T2) *A. flavus* with an overall variance of 28.47% (PLS1 = 17.92% and PLS2 = 10.55%), high predictive ability (Q2Y = 0.86), goodness of fit (R2X = 0.51 and R2Y = 0.98), and considerable significance metric (*p* = 0.0003), as indicated in Fig. 3b. We selected the significantly discriminant metabolites contributing maximally to the observed variance in the metabolite profiles of VOC treated *A. flavus* based on the variable importance in projection (VIP) > 0.7 and *p* < 0.05 in the respective PLS-DA model (Table 1). Altogether, we observed 32 significantly discriminant secondary metabolites falling under different chemical classes, including alkaloid (1), xanthone (2), coumarin (3), anthraquinones/ quinones (4–9), indole/ indole-terpenes (10–12), fatty acid derivatives/ oxylipins (13–18), miscellaneous compounds (19–21), and the non-identified metabolites (22–32). Considering the importance of secondary metabolites in shaping the fungal trophic interactions and stress response, we quantitatively expressed their time correlated relative abundance using the heat map (Fig. 3c). Notably, we observed an approximately two-fold higher relative abundance of polyketide (PK) mycotoxin, aflatoxin B1, and its precursors compounds (5-methoxysterigmatocystin, versicolorin A, and averufin except versicolorin B) for 7-day incubated *A. flavus* subjected to 1-Octen-3-ol gradient treatments (T2). However, the relative abundance of non-ribosomal peptide (NRP) mycotoxins including α-cyclopiazonic acid and paxilline decreased considerably in both the treatment groups T1 and T2 on 7-day. Further, some of the anthraquinone compounds, including asparasone A, displayed lower relative abundance in VOC- treated groups (T1 and T2) compared to the untreated samples of *A. flavus* on 7-day.

**Table 1:** List of the significantly discriminant metabolites selected using the PLS-DA model (VIP > 0.7 and *p* < 0.05) based on the UHPLC–LTQ–Orbitrap–MS datasets for *A. flavus* extracts following the VOC treatments.

No.	RT (min)	Tentative metabolites	[M + H]^+^	[M − H]^‒^	M.W	Elemental composition	Error (ppm)^a^	MS^n^ fragment pattern	*p* value	References
**Alkaloid**										
1	5.63	Aspergilline C	441.2026	439.1951	440	C_24_H_29_N_2_O_6_ (+)	1.353	(+) 441 > 291, 265 > 177	5.00E−06	Uka et al*.*^50^
**Xanthone**										
2	5.86	5-Methoxysterigmatocystin	355.0819	353.0728	354	C_19_H_15_O_7_ (+)	1.917	(−) 353 > 338 > 323, 310 > 295, 279	1.23E−03	Carvajal-Campos et al*.*^51^
**Coumarin**										
3	6.12	Aflatoxin B1	313.0710	311.0619	312	C_17_H_13_O_6_ (+)	1.007	(+) 313 > 285 > 270, 257 > 253, 242	2.39E−02	Carvajal-Campos et al*.*^51^
**Anthraquinones/ quinones**										
4	6.55	Asparasone A	381.0583	357.0673	358	C_18_H_14_O_8_Na (+)	0.686	357 > 339, 299	3.00E−05	Carvajal-Campos et al*.*^51^ and Malysheva et al*.*^52^
5	6.72	Anthraquinone derivative 1	269.0804	267.0796	268	C_16_H_13_O_4_ (+)	− 1.804	267 > 252 > 223, 208 > 195	3.90E−05	Fouillaud et al*.*^53^
6	7.10	Anthraquinone derivative 2	‒	315.0555	316	C_16_H_11_O_7_ (‒)	5.409	315 > 297 > 269, 253 > 241, 225	0.00E+00	Fouillaud et al*.*^53^ and Carvajal-Campos et al*.*^51^
7	5.56	Chrysophanol	255.0657	253.0551	254	C_15_H_11_O_4_ (+)	1.743	(+) 255 > 237, 227, 199 > 181, 171 > 153 (−) 253 > 253, 224, 209, 185	1.00E−06	Tripathi et al*.*^54^
8	7.26	Versicolorin B	341.0656	339.0558	340	C_18_H_13_O_7_ (+)	0.178	339 > 321, 311, 297 > 295, 269, 253	0.00E+00	Carvajal-Campos et al*.*^51^
9	7.94	Versicolorin A	339.0504	337.0410	338	C_18_H_11_O_7_ (+)	1.330	337 > 309 > 281, 265 > 252	1.71E−02	Carvajal-Campos et al*.*^51^
**Indole/ Indole terpenes**										
10	7.61	Sulpinine C	536.3008	534.2937	536	C_32_H_42_NO_6_ (+)	− 1.258	534 > 476 > 416 > 400, 358	6.50E−05	Laakso and Gloer^55^
11	8.13	α-Cyclopiazonic acid (CPA)	337.1545	335.1450	336	C_20_H_21_N_2_O_3_ (+)	− 1.095	335 > 180, 154, 140	1.10E−03	Uka et al*.*^50^
12	8.72	Paxilline	436.2480	434.2399	435	C_27_H_34_NO_4_ (+)	− 0.080	434 > 415, 346 > 331, 316, 302	2.70E−05	Carvajal-Campos et al*.*^51^
**Fatty acid derivatives/ oxylipins**										
13	6.44	9,12,13-TriHOME	353.2305	329.2383	330	C_18_H_34_O_5_Na (+)	1.938	(−) 329 > 311 > 293, 229, 211, 199, 171	8.60E−05	Singh and Lee^13^ and Son et al*.*^56^
14	7.04	Fatty acid derivative	295.2262	293.2164	294	C_18_H_31_O_3_ (+)	− 1.834	(−) 293 > 275, 235 > 231, 177	1.05E−02	CHCD
15	7.51	5,8-DiHODE	335.2192	311.2270	312	C_18_H_32_O_4_Na (+)	− 3.483	(−) 311 > 293, 275, 173 > 249, 231	1.79E−03	Singh and Lee^13^
16	7.84	12,13-DiHOME(9)	337.2350	313.2433	314	C_18_H_34_O_4_Na (+)	− 2.899	(−) 313 > 295 > 277, 259, 251, 233	0.00E+00	Singh and Lee^13^; Standard compound
17	8.00	Dihydroxy-octadecadienoic acid (DiHODE)	335.2191	311.2274	312	C_18_H_32_O_4_Na	− 0.479	(−) 311 > 293, 275, 211, 187, 171, 157	4.10E−05	Son et al*.*^56^; CHCD
18	9.41	Fatty acid derivative	297.2422	295.2322	296	C_18_H_33_O_3_ (+)	− 0.678	(−) 295 > 277, 251 > 233, 179, 165, 139	0.00E+00	CHCD
**Miscellaneous**										
19	7.75	Aspergilone A	387.1931	431.1896	386	C_26_H_27_O_3_ (+)	− 6.176	(+)387 > 331 > 275, 231, 175	0.00E+00	Shao et al*.*^57^
20	7.84	Aszonapyrone A	457.2947	455.2870	456	C_28_H_41_O_5_ (+)	− 0.242	455 > 437, 411, 393 > 375, 325, 287, 269	2.23E−04	Kimura et al*.*^58^
21	9.16	Averufin	391.1653	367.0869	368	C_20_H_15_O_7_ (‒)	4.696	367 > 349, 323, 267 > 223	2.00E−06	KNApSAcK core system
**Non-identified**										
22	5.14	N.I. 1	445.2339	443.2264	444	C_24_H_33_N_2_O_6_ (+)	0.464	445 > 267, 250 > 179, 137	2.00E−06	‒
23	5.20	N.I. 2	246.1129	244.1025	245	C_14_H_16_NO_3_ (+)	1.748	246 > 228 > 200 > 183, 133	4.00E−06	‒
24	6.47	N.I. 3	525.2715	523.2636	524	C_27_H_41_O_10_ (+)	4.048	523 > 417 > 311 > 267	0.00E+00	‒
25	7.17	N.I. 4	548.2863	546.2801	547	C_29_H_42_NO_9_ (+)	1.663	546 > 417, 311	2.36E−04	‒
26	7.77	N.I. 5	‒	339.0558	‒	C_11_H_15_O_12_ (‒)	− 3.183	‒	2.78E−04	‒
27	7.98	N.I. 6	532.2917	530.2847	531	C_25_H_42_N_2_O_10_ (+)	− 0.365	530 > 401 > 383, 357, 285 > 203, 179	1.04E−04	‒
28	9.25	N.I. 7	581.2431	579.2350	580	C_23_H_42_O_15_Na (+)	− 0.238	579 > 523, 417 > 347, 149	0.00E+00	‒
29	9.45	N.I. 8	540.4253	538.4178	539	C_31_H_58_NO_6_	− 1.841	538 > 519, 494, 450, 408, 337, 355	4.16E−04	‒
30	10.40	N.I. 9	669.3312	667.3269	668	C_41_H_47_O_8_ (‒)	− 0.377	667 > 637 > 619, 431, 369 > 351, 267, 225	2.05E−03	‒
31	10.59	N.I. 10	637.3049	681.3083	636	C_27_H_47_N_3_O_14_ (+)	− 0.415	681 > 653 > 447 > 285 > 267	2.24E−03	‒
32	11.25	N.I. 11	637.3063	635.2993	636	C_27_H_47_N_3_O_14_ (+)	− 0.415	635 > 367 > 337, 267, 149	0.00E+00	‒

RT: Retention time for chromatographic elution. CHCD: Combined Chemical Dictionary, Chapman and Hall, London, UK, 1992.

^a^Mass tolerance from elemental composition analysis.

We observed marked variations in the relative abundance of oxylipin compounds following the growth and morpho-transformation changes in response to the VOCs treatments. Following the 7-day of incubation, all oxylipin compounds except 9,12,13-trihydroxy-10E-octadecenoic acid (9,12,13-TriHOME) were significantly reduced for VOC treated groups (T1 and T2) compared to the untreated sets. Intriguingly, *A. flavus* exposed to the lower titers of 1-Octen-3-ol (T2; 100 and 200 ppm/day) displayed approximately two-fold higher relative abundance of oxylipins compared to those exposed to its higher titers (T2; 400 ppm/day) as well as the *A. oryzae* volatilome (T1) on 9-day. The patterns of observed variation in the relative abundance of oxylipin compounds coupled with reduced sclerotia formation (Fig. 2b) suggests their intricate role in modulating *A. flavus* response to 1-Octen-3-ol gradient treatments.

Further, aspergilone A and the non-identified (N.I) metabolites except N.I. 3 and N.I. 5 were observed more abundant in the VOCs treated (T1 and T2) *A. flavus* compared to the untreated samples. These trends were coupled with the reduced growth rates and sclerotial counts in the respective treatments on 7-day of incubation. However, we observed reverse trends for the 9-day incubated samples with most of the uncharacterized metabolites (N.I. 4 and N.I. 7–11) being less abundant in *A. flavus* subjected to T2 as compared to T1, which suggests the distinctive effects of standard 1-Octen-3-ol gradients and *A. oryzae* volatilome with a blend of C-8 VOCs.

### Correlations among the 1-Octen-3-ol exposure treatments, developmental phenotypes, and metabolite profiles for *A. flavus*

**Figure 4:**
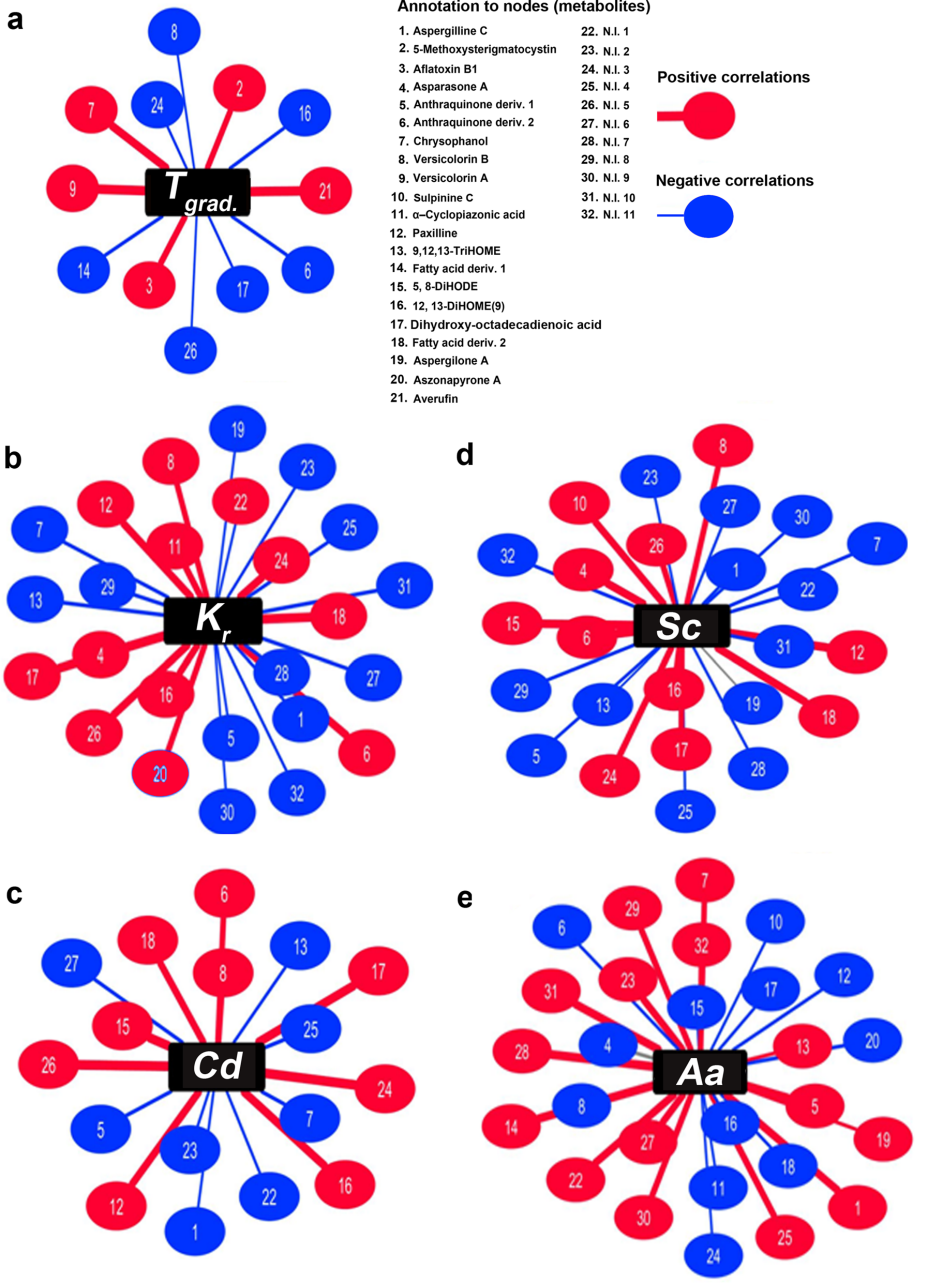
The spring-embedded network indicating the individual correlations between the significantly discriminant secondary metabolite levels and (**a**) the 1-Octen-3-ol gradient treatments, *T*_*grad.*_ (**b**) mycelial growth rates, *K*_*r*_ (**c**) conidial density, *Cd* (**d**) sclerotial counts, *Sc* and (**e**) secreted α-amylase activity, *Aa* for *A. flavus* subjected to varying VOC treatments. The nodes of the network represent the metabolites indicated with unique codes while the edges display the significant correlations (Pearson's coefficient *r*, 0.4 > and <  − 0.4 at *p* < 0.01) for multiple testing. The positive and negative correlations are indicated with red and blue colored nodes, respectively.

We argue that the controlled exposure of *A. flavus* to the 1-Octen-3-ol sources might have influenced its developmental phenotypes (growth, conidiation, sclerotia morphogenesis, and extracellular secretion of amylases) coupled with subtle metabolite levels. Hence, we evaluated the statistical correlation networks (Pearson's coefficient r, 0.4 > and <  − 0.4; *p* < 0.01) among the 1-Octen-3-ol treatments, the *A. flavus* phenotypes, and the associated metabolite abundance, individually in each case (Fig. 4). Notably, the exposure to 1-Octen-3-ol gradients to *A. flavus* was positively correlated with mycotoxins production including aflatoxin B1 and its precursors (5-methoxysterigmatocystin, versicolorin A, averufin), and an anthraquinone compound chrysophanol. Further, we observed negative correlations between the 1-Octen-3-ol treatments and the relative abundance of oxylipin compounds (Fig. 4a). Intriguingly, the higher relative abundance of PK mycotoxins coupled with the lower levels of oxylipin compounds concurred with the reduced growth rates and sclerotia formation in VOC treated *A. flavus*, most notably on 7-day.

Focusing on *A. flavus* developmental phenotypes following the VOC treatments, the radial growth rates (*K*_*r*,_ mm^2^/day) displayed significantly positive correlations with certain anthraquinone compounds (asparasone A and anthraquinone derivative 2), indole-terpene NRP mycotoxins (α-cyclopiazonic acid and paxilline), oxylipin compounds (12,13-dihydroxy-9-octadecenoic acid (12,13-DiHOME) and dihydroxy-octadecadienoic acid), a quinone compound (versicolorin B), and a meroditerpene alkaloid (aszopyrone). Conversely, the negative correlations were evident between mycelial growth and aspergilline C (a CPA-derived NRP mycotoxin), chrysophanol (antraquinone), oxylipin 9,12,13-TriHOME, and aspergilone A (di-terpenoid) as displayed in Fig. 4b. The mycelial transformation to sclerotia displayed strong positive correlations with most oxylipin compounds except 9,12,13-TriHOME suggesting their intertwined functional role in *A. flavus* development and morpho-transformation (Fig. 4c). In addition, positive correlations were evident between sclerotia formation and its characteristic pigment compound, i.e., asparasone A (anthraquinone), which imparts dark-brown coloration during their later stages of development^20^. Further, positive correlations were also observed for an anthraquinone compound, a quinone aflatoxin precursor (versicolorin B), and an NRP-derived indole terpene mycotoxin (paxilline). On the other hand, sclerotia formation showed negative correlations with most of the anthraquinone compounds (except asparasone) including aspergilline C and aspergilone A as well as the non-identified (N.I.) metabolites. The conidial density variation in VOC treated *A. flavus* (Fig. 4d) displayed strong positive correlations with oxylipin compounds (5,8-dihydroxy-9,12-octadecadienoic acid (5,8-DiHODE), 12,13-DiHOME, and dihydroxy-octadecadienoic acid) in agreement to the previous reports suggesting their functional roles^21,22^. However, negative correlations were observed between conidia development and an oxylipin compound 9,12,13-TriHOME, anthraquinone compounds (derivative 1 and chrysophanol), and the indole terpene mycotoxins (paxilline and aspergilline C).

The extracellular secretion of hydrolytic enzymes and biosynthesis of secondary metabolites, especially the mycotoxins, analogously complement the *A. flavus* colonization on host surfaces^23^. We observed that the trends of amylase activity in VOC treated *A. flavus* showed positive correlations with selected secondary metabolites including aspergilline C, certain quinone/anthraquinones (chrysophanol and derivative 1), oxylipins (9,12,13-TriHOME and fatty acid derivative 1), diterpenoid compound aspergillone A, and most of the N.I compounds (Fig. 4e). Conversely, negative correlations were evident between the secreted amylase activity and quinone/anthraquinones (derivative 1, asparasone A, and versicolorin B), indole terpenes (sulpinine C, CPA, and paxilline), some oxylipin compounds (5,8-DiHODE and DiHODE), and meroditerpene compound aszonapyrone A.

## Discussion

The VOCs mediated interactions among the *Aspergillus* species under section *Flavi* including those between *A. flavus* and *A. oryzae* are conserved as they colonize the common niche environment ranging from field crops to fermented foods^24–26^. Hence, we examined the effects of *A. oryzae* volatilome, narrowing down to infochemical compound 1-Octen-3-ol, on toxicogenic *A. flavus* strain using an indigenously designed twin-plate assembly. First, we sought to identify the C-8 VOCs and evaluated their time-correlated relative abundance in the *A. oryzae* headspace that may have influenced the developmental and metabolic changes in *A. flavus*. The biosynthesis of C-8 VOCs is associated with the enzymatic oxidation and lysis of linoleic acid which influence the fungal development through the production of short-chain functional oxylipins^12,27,28^. Herein, we observed the highest relative abundance of 1-Octen-3-ol followed by 2-Octenal, and 3-Octanone among others in the *A. oryzae* headspace volatilome. Considering the significantly higher proportions of 1-Octen-3-ol in *A. oryzae* volatilome and its role in *Aspergillus* development, we explored it potential signaling functions in mediating *‘A. oryzae – A. flavus’* interactions^10–12,29,30^. We observed a concentration dependent decrease in mycelial growth of *A. flavus* following the 1-Octen-3-ol exposure treatments, most notably on 7-day of incubation (Figs. 1b, 2a, and Supplementary Fig. S1). These results are congruent to the previous reports suggesting the fungicidal (~ 50–100 ppm) as well as fungistatic (~ 5–10 ppm) effects of 1-Octen-3-ol on Ascomycetes fungi, *Pseudogymnoascus destructans*, while cultivation in partitioned bi-petri plates^31^.

In some *A. flavus* strains, the mycelial growth is followed by the onset of two developmental phenomena namely sclerotia morphogenesis and conidiation. These two stages of *A. flavus* development are believed to be reciprocally regulated by common regulatory genes, *VeA* and *LaeA*^15,20^. Accordingly, we observed lesser sclerotial counts coupled with higher conidial density for *A. flavus* subjected to 1-Octen-3-ol gradient exposure (T2_*100-200* *ppm/*day_) on 7-day, compared to the untreated strains (Figs. 1c, 2b). Further, we observed an inverse relation between the increasing titers of 1-Octen-3-ol and the total sclerotial counts, suggesting the morpho-transformation inhibitory effects of the C-8 VOCs, in agreement to our previous study^13^. Although an inverse correlation was valid for 1-Octen-3-ol gradient treatments and morpho-transformation on 9-day, a marked increase (~ 150–300%) in sclerotial counts suggests both the *A. flavus* resilience as well as the transient nature of 1-Octen-3-ol impact. Notably, the lowest sclerotial counts were observed for *A. flavus* exposed to *A. oryzae* volatilome which suggests the synergistic effects of C-8 VOC blends.

Evaluating the effects of VOC treatments (T1 and T2) on conidiation, we observed lower conidial density for *A. flavus* subjected to higher titers (400 ppm/day) of 1-Octen-3-ol and *A. oryzae* volatilome compared to the untreated samples. In contrast, significantly higher conidial density (*p* < 0.01) values were evident for the strains treated with lower titers (≤ 100–200 ppm/day) of the standard VOC. Hence, we suggest a ‘critical threshold titer (*x*)’ at which the *A. flavus* exposure to the 1-Octen-3-ol gradients (≤ 200 ppm/day, *x*, ≥ 400 ppm/day) may induce ambiguous effects on conidiation pattern (Figs. 1d, 2c). Previously, Chitarra et al.^10,11^ have suggested that 1-Octen-3-ol may reversibly induce microcycle conidiation or conidia budding through circumventing their hyphal transformation to conidiophore formation stage in *Penicillium paneum*. However, the 9-day incubated untreated strains displayed higher conidial density as compared with those subjected to VOC treatment, which suggests the transient and time-correlated nature of 1-Octen-3-ol mediated effects on *A. flavus* development. We argue that the twin-plate exposure to sub-critical threshold titers (*x ≤ *200 ppm/day) of 1-Octen-3-ol may reversibly alter the developmental shift in *A. flavus* toward microcycle conidiation.

The saprophytic growth of *A. flavus* in its ecological niche is facilitated by secretory hydrolases, where the α-amylase mainly degrades the host tissue polysaccharides and thus influence the fungal trophic interactions^19,32^. Considering the *Aspergillus* development, the secretion of amylases is interfered by the higher conidiation rates, where the two phenomena are reciprocally regulated by a cascade of genes including *flbA*,* brlA*, and *fluG*^33^. However, we observed a significantly higher α-amylase activity coupled with higher conidial density in *A. flavus* subjected to 1-Octen-3-ol exposure at sub-critical threshold titers (*x* ≤ 200 ppm/day). The observed anomaly can be attributed to the simultaneous induction of enhanced membrane permeability and microcycle conidiation by 1-Octen-3-ol treatments, as suggested previously by Chitarra et al.^10,11^. However, the *A. flavus* exposure to critical threshold titers (*x* ≥ 400 ppm/day) of 1-Octen-3-ol affected lower conidial density coupled with higher amylase secretion compared to the untreated sets (Fig. 2d). We hypothesize that the critical threshold titers of 1-Octen-3-ol may reversibly arrest the *A. flavus* development (growth, morphogenesis, and conidiation), which prompts fungi to enhance hydrolytic enzymes secretion for sequestering more nutrients. Previously Hu et al*.*^34^ have pointed out that *A. flavus* deletion mutant for some transcription factor (ΔRum1) displays epigenetic perturbations which modulates altered sclerotia-conidia balance, coupled with enhanced amylase secretion and aflatoxicosis.

An increasing number of studies have emphasized that microbial interaction including those mediated by signaling VOCs may trigger the cryptic biosynthetic pathways shaping their chemical ecology^35–37^. Especially, the mycotoxins are increasingly been recognized as 'fitness factors' influencing the fungal virulence and hence their ecological interactions in nature^38^. In the present study, we observed a marked disparity in the relative abundance of secondary metabolites for *A. flavus* subjected to VOC treatments, as displayed in Fig. 3. It is most noteworthy that 1-Octen-3-ol treatments significantly enhanced PK mycotoxin, aflatoxin B1, and its precursor compound biosynthesis in the sclerotia forming strain of *A. flavus* (Fig. 3c). The spring embedded correlation map analyses unraveled strong positive correlations between the 1-Octen-3-ol treatments and polyketide mycotoxin compounds (Fig. 4a). Recently, Pennerman et al*.*^29^ have also reported the upregulated production of PK mycotoxin 'patulin' in *P. expansum* subjected to sub-inhibitory levels of 1-Octen-3-ol exposure, which suggest its potential signaling function to induce the stress response in fungi. We propose that the heightened mycotoxin production determines the competitive fitness of interacting species where the potential ammensalic interactions are being shaped. The C-8 VOCs reportedly affected the oxidative stress condition on interacting species which may lead to heightened aflatoxin B1 production through various mechanisms^39^. However, the terpenoid derived NRP mycotoxins including α-cylopiazonic acid (CPA) and paxilline displayed significantly lower abundance in VOC treated (T1 and T2) sets, which suggests a selective metabolic response of *A. flavus* to the specific VOC (Fig. 3c). Another important class of secondary metabolites perturbed following the VOC treatments (T1 and T2) were anthraquinones, which are directly linked with the developmental phenotypes including growth, conidiation, and sclerotia formation in fungi (Figs. 3, 4). Functionally, the anthraquinone derivative ‘asparasone A’ is important for sclerotia pigmentation, which marks an important step in the life cycle of sclerotia forming fungi^21,40^.

The fungal oxylipin constitutes a wide spectrum of short chain (~ C_6_ – C_9_) volatiles compounds and the long chain (~ C_16_—C_20_) secreted molecules produced through LOX (lipoxygenase) activity, which altogether modulates its quorum sensing mechanism and associated development^22,41^. In particular, the linoleic acid (C18:2) derived oxylipins are known to regulate a balance between ‘conidia-sclerotia’ formation depending on cell density in *A. flavus* cultures^42^. Similarly, the C-8 volatile oxylipin ‘1-Octen-3-ol’ is reported to induce quorum sensing under high conidial density conditions in *Penicillium* species, inhibiting its normal course of conidia germination^10–12^. Herein, a lower relative abundance of most oxylipin compounds except for 9,12,13-TriHOME was evident for VOC-treated *A. flavus* on 7-day of incubation (Fig. 3c). However, strong positive correlations were evident between the oxylipin abundance and *A. flavus* phenotypes (Fig. 4b–d) being altered following the VOC treatment, which signifies their pivotal role in modulating *Aspergillus* development. Further, we observed a number of uncharacterized (N.I) metabolites as well as the miscellaneous ones showing marked perturbation and statistical correlations with developmental phenotypes in VOC treated *A. flavus* (Figs. 3c, 4). In particular, the di-terpenoid derivatives including aspergilone A and sulpinine C, meroterpenoid derivative aszopyrone A, CPA derivative aspergillin C, and anthraquinone compound chrysophanol have largely uncharacterized functions during the *Aspergillus* development.

In the present study, we propose that the critical threshold titers of 1-Octen-3-ol in the *A. oryzae* headspace may elicit a developmental response in *A. flavus* characterized by reduced growth, delayed morpho-transformation, and altered conidiation patterns. Intriguingly, the attenuated development in *A. flavus* was coupled with the heightened production of secretory α-amylases and PK mycotoxins. We examined a few secondary metabolite classes including alkaloids, coumarins, anthraquinones, indole terpenoids, and oxylipins perturbed following the VOC treatment in *A. flavus*. The present study illustrates how the gradient treatment with C-8 VOC ‘1-Octen-3-ol’ may influence interspecies *Aspergillus* interactions through providing an insight into the metabolomic shift in *A. flavus*. Although these interactions are hard to extrapolate for complex microbiomes in environment, we can hypothesize such interactions in relatively simple semi-natural environs like fermented foods, stored commodities, laboratory cultivations systems, or even the indoor building environs^43–45^. Herein, we determine the potential role of 1-Octen-3-ol in modulating the *A. flavus* development and its inter-species interactions which may help in designing the effective strategies toward aflatoxin management.

## Methods

### Chemicals

Analytical grade (≥ 98.0%) R enantiomer of 1-Octen-3-ol standard compound was purchased from Sigma-Aldrich). Solvents including HPLC grade acetonitrile, methanol, ethyl acetate, hexane, and di-chloromethane were purchased from Fisher Scientific (Waltham, MA, USA).

### Fungal cultures and cultivation

The *Aspergillus flavus* KCCM 11899 and *A. oryzae* KCCM 60345 were procured from the 'Korean Culture Center of Microorganisms (KCCM)' at the National Academy of Agricultural Sciences, Seodun-dong, Suwon, Republic of Korea. The fungi were sub-cultured and maintained (14 days, 30 °C) in malt-extract agar (MEA) for harvesting fresh spores toward inoculation. A fixed conidia suspension of 10 μL (1 × 10^6^ spores/mL) was centrally inoculated in WATM agar for all experimental and control sets^13^. The conidia counting in all steps of experiment was performed using Neubauer haemocytometer.

### Headspace—solid phase microextraction (HS-SPME) for *A. oryzae* (P2—source of VOCs)

We harvested the mycelial biomass for 7-day and 9-day incubated *A. oryzae* samples from plate 2 (treatment 1) in Ringer's solution (10 mL), using the method partially adapted from Singh and Lee^13^ and Costa et al*.*^46^. The freshly harvested samples were added with sodium chloride (0.2 g/mL) toward better extraction of VOCs through reducing their solubility in Ringer's solution. Standard compound 'linalool' (1 ppm) was added as the internal standard (IS) and the harvested samples were transferred to 20 mL SPME vial (Merck KGaA, Germany) with septum closed. At this step, the freshly harvested biomass was further incubated for 1 h under the experimental conditions so that VOCs from the physiologically active culture may accumulate in closed SPME vials. Three biological replicates of *A. oryzae* were subjected to headspace (HS) VOC extraction using divinylbenzene/carboxen/polydimethylsiloxane (DVB-CAR-PDMS) StableFlex (1 cm) fiber (Sigma-Aldrich) for 60 min at 65 °C with intermitted agitation (100 rpm) on L-PAL3 GC autosampler (LECO Corporation, St. Joseph, MI, USA).

We maintained three biological replicates for each of the sample harvested. The absolute quantities of 1-Octen-3-ol were determined using the linear least square regression analysis of the standard curve (linear range 0.5–6.5 ppm) with three analytical replicates. The standard compound gradients were first added in 10 mL Ringer's solution with NaCl (0.2 g/mL) and subjected to the same extraction steps as described for the *A. oryzae* volatilome.

### *A. flavus* exposure to *A. oryzae* volatilome (treatment 1) and the standard VOC gradients (treatment 2)

**Figure 5:**
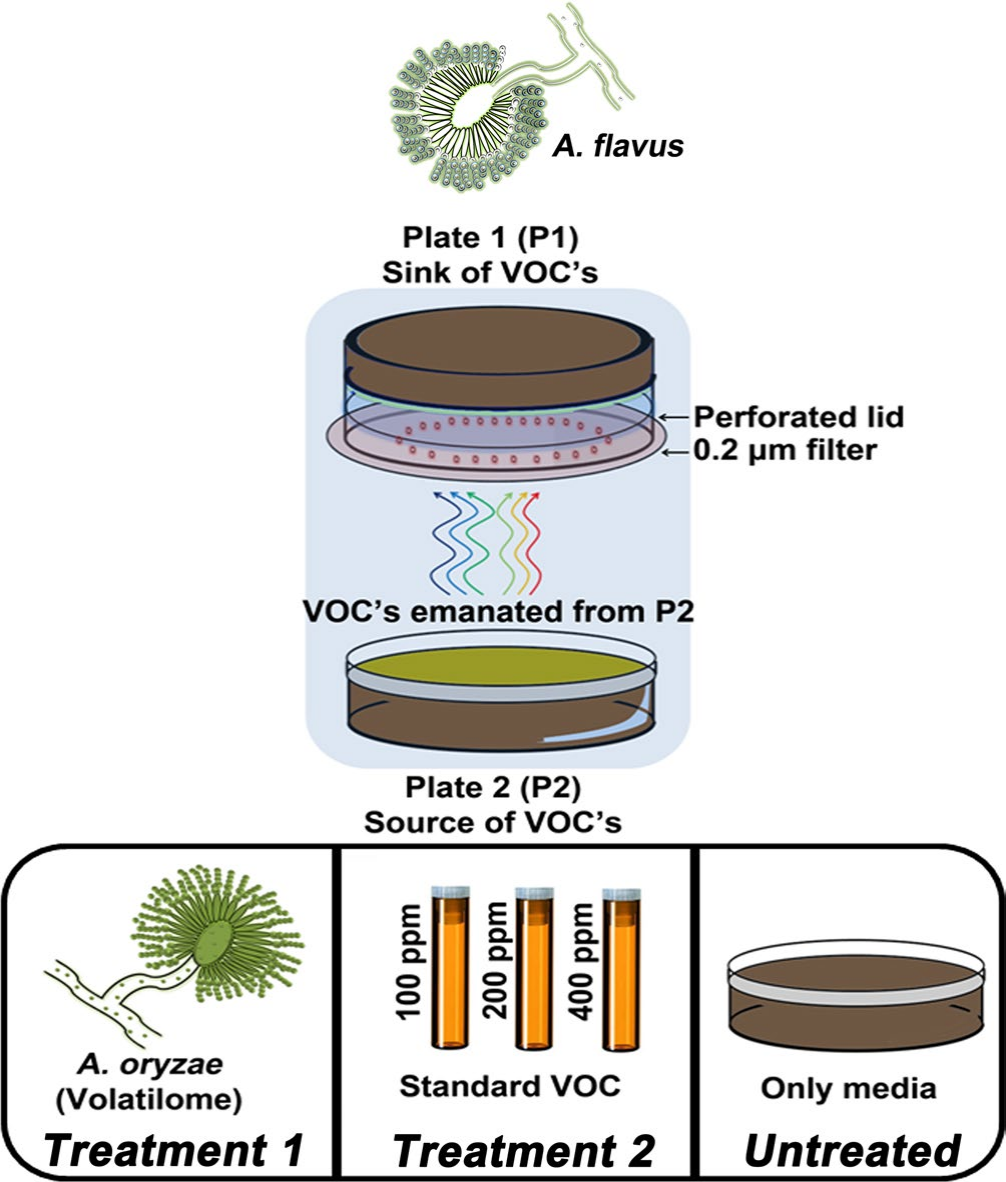
Schematics displaying the design of experiment (DOE) to examine the effects treatment 1 (T1—*A. oryzae* volatilome) and treatment 2 (T2—1-Octen-3-ol gradients) on *A. flavus* in twin-plate experiment (P1 × P2). The effects of treatments (T1 and T2) on *A. flavus* were compared with untreated set serving as the experimental control to normalize the baseline effects of endogenously produced VOCs. All treatments and control sets were examined maintaining the three biological replicates of each.

The effects of *A. oryzae* volatilome and the varying gradients of standard compounds on *A. flavus* development were examined using the twin-plate (P1 × P2) experiment (Fig. 5), previously designed and reported by Singh and Lee^13^. The plate 1 (P1—sink of VOCs) with perforated lid and extrinsic filter paper was inoculated with *A. flavus* and assembled face-to-face with plate 2 (P 2—source of VOCs).

In treatment 1, we examined the pair-wise *Aspergillus* interactions through inoculating P2 with *A. oryzae*. The choice of *A. oryzae* as the partner strain as well as the source of natural volatilome was inspired by the reports describing the untoward colonization of fermented foods by the toxicogenic strains of *A. flavus*^24–26^. In treatment 2, the selective effects varying concentration gradients of 1-Octen-3-ol on *A. flavus* were examined using the parallel experimental sets. Briefly, following the 4 day incubation period necessary to acquire competence in *A. flavus* (P1), the P 2 of twin-plate was infused with varying gradients of standard 1-Octen-3-ol (100 ppm/day, 200 ppm/day, and 400 ppm/day). Another reason rationalizing the timing of standard VOC infusion was the reported onset of C-8 VOCs production in *Aspergillus* species after 3–4 days of incubation under aerobic conditions^47^. We maintained the experimental controls for treatment 1 and 2 by incubating *A. flavus* (P1) under the untreated conditions without any VOCs sources in P2 of twin-plate. Herein, we wish to emphasize that the untreated set will serve as the control toward observing the baseline effects of endogenously produced C-8 VOCs on the developmental phenotypes of *A. flavus* itself. Following the inoculation, all twin-plates were placed flat ensuring P1 (*A. flavus*) at the top and P2 (*A. oryzae* in T1 and standard compounds in T2) lay at the bottom, allowing the upward flow of low vapor pressure VOCs from P2 to P1. We maintained three biological replicates for each of the treated (T1 – *A. oryzae* volatilome and T2 – 1-Octen-3-ol gradients) and untreated sample, all incubated at 30 °C under dark conditions for 7- and 9-days.

### VOC analyses based on Gas chromatography–time of flight–mass spectrometry (GC–TOF–MS)

The VOCs were analyzed on an Agilent 7890A system (Agilent, Santa Clara, CA, USA) coupled with Pegasus HT TOF–MS and L-PAL3 GC autosampler (Leco Corporation, St. Joseph, MI, USA). The VOCs separation was performed on RTx-5MS fused silica capillary column (30 m × 250 μm i.d., pore size 0.25* μm*) purchased from J&W Scientific, Folsom, CA, USA with helium as the carrier gas at the constant flow rate of 1.5 mL/min. The extraction fiber was desorbed in GC injection port at 270 °C for 3 min and the analysis was performed in splitless mode. The column temperature was initially maintained at 45 °C for the first 9 min, ramped to 85 °C in 4 min and maintained for 3 min, further elevated to 120 °C in 2.5 min and maintained for 2 min, and finally elevated to 270 °C in 7.5 min and maintained as such for the final 2 min. The overall chromatographic run program spanned 30 min. The MS data was recorded at the rate of 100 scans per second in the m/z range of 35–300. The variance across different steps of metabolic profiling was assessed using pooled quality control (QC) sample runs intermittently. The VOCs examined using GC–TOF–MS were characterized using the standard compound comparing their RT and MS fragments. In addition, we confirmed the spectral data for VOCs with available databases including VocBinBase^48^ and National Institute of Standards and Technology (NIST), ver. 2.0, 2011, FairCom, USA.

### Sample harvest and analyses of developmental phenotypes for *A. flavus*

The VOC treated and untreated sets of *A. flavus* were harvested following 7- and 9-days of incubations under twin-plate experiment. The *A. flavus* samples were immediately analyzed for growth and developmental phenotypes including radial growth rates (*K*_*r*_), sclerotial counts (*Sc*), conidial density (*Cd*). Further, the fungal biomass was harvested for extracellular enzyme extraction and assayed for α-amylase activity using the protocol adapted from Chancharoonpong et al*.*^49^ and Singh and Lee^14^. The remaining biomass was stored under deep freezing conditions (− 80 °C), until further analyses.

### Metabolite extraction

The *A. flavus* cultures harvested from P1 were quenched with liquid nitrogen and pulverized using pestle and mortar. The fine ground fungal biomass was subjected to metabolite extraction using the solvent mixture consisting of methanol, hexane, dichloromethane, and ethyl acetate (1:1:2:3) with 1% formic acid, while formononetin (5 mg/L) was used as the internal standard (IS). The pulverized fungal biomass was added with 20 mL of extraction solvent and the resulting mixture was subjected to overnight agitation (200 rpm) at 30 °C. The samples were sonicated for 1 h and centrifuged (10,000 × g) for 10 min to collect the supernatant. The sample supernatants were collected in scintillation vials (20 mL) and dried under speed vacuum concentrator (Hanil Scientific, Korea). The dried samples were re-suspended in extraction solvent at appropriate concentrations (10 mg/mL or 10,000 ppm) prior to the chromatographic analyses.

### Metabolite profiling based on ultrahigh performance liquid chromatography–linear trap quadropole–orbitrap–mass spectrometry (UHPLC–LTQ–Orbitrap–MS)

The metabolite extracts from *A. flavus* subjected to varying VOCs treatment conditions in P1 of twin-plate assembly were examined using UHPLC-LTQ-Orbitrap-MS system coupled with Vanquish binary pump H system (Thermo Fisher Scientific, Waltham, Massachusetts, USA). The reverse phase chromatographic separation of metabolites was performed on Phenomenex KINETEX C18 column (100 mm × 2.1 mm, 1.7 μm particle size; Torrance, CA, USA). The mobile phase composed of water (solvent A) and acetonitrile (solvent B), were each added with 0.1% formic acid, and the flow rate was maintained at 0.3 mL/min. The 14 min gradient run program commenced with 5% solvent B for 1 min followed by its linear increase to 100% in next 9 min, maintained for 1 min, and re-equilibrated to initial condition (5% solvent B) in the final 3 min. The sample injection volume was 5 μL and the column temperature was maintained at 40 °C. The tandem MS was performed on LTQ-Orbitrap-Velospro with ion-trap (IT) MS and heated ESI or HESI-II probe (Thermo Fisher Scientific). The MS parameters were fixed at probe heater temperature of 300 °C, capillary temperature of 350 °C, and the capillary voltages of 2.5 kV (− ESI) and 3.7 kV (+ ESI). The metabolites examined using UHPLC-MS were characterized through comparing multiple parameters like retention time (RT), molecular weight (M.Wt.), elemental composition (molecular formula), and associated m/z fragmentation patterns with those retrieved comprehensively from standards, in house library, available databases, and published literatures.

### Data processing and statistical analyses

The raw data files obtained from UHPLC-MS and GC–TOF–MS analyses were converted to NetCDF (network Common Data Form) file formats with *.cdf* extension using corresponding software packages. The files (*.cdf*) were preprocessed for peak list alignment, peak detection, RT, normalized peak intensities, and accurate masses comparing their full scan nominal mass using MetAlign software. The aligned data was further subjected to multivariate analyses to evaluate the class-wise variance in datasets and determining the significantly discriminant metabolites (VIP > 0.7, *p* < 0.05) using SIMCA-P + (version 12.0, Umetrics, Umea, Sweden). The quantitative data for phenotype analyses were subjected to pair-wise comparison based on ANOVA and Duncans/ Tukey multiple range tests using PASW statistica 18 software (SPSS Inc. Chicago, Illinois, USA). The interaction networks between varying metabolite levels and phenotypes were visualized using Cytoscape software (v3.7.2).

## Supplementary information

Supplementary Information.

## Data Availability

The data in this study are available from the corresponding author upon reasonable request.
