# Supplementary material for: Critical thresholds of 1-Octen-3-ol shape inter-species Aspergillus interactions modulating the growth and secondary metabolism
Source: Sci Rep. 2020 Jul 6;10:11116. doi: 10.1038/s41598-020-68096-x (PMC7338521; doi:10.1038/s41598-020-68096-x)
Supplement: Supplementary file 1 — Supplementary Information. [file 41598_2020_68096_MOESM1_ESM.docx]

**Critical thresholds of 1-Octen-3-ol shape inter-species *Aspergillus* interactions modulating the growth and secondary metabolism**

**Digar Singh, Su Young Son, Choong Hwan Lee^*^**

Department of Bioscience and Biotechnology, Konkuk University, 05029, Seoul, Korea

***** Corresponding author: Telephone: (+82) 220496177; Fax Number: (+82) 24454291; E-mail: chlee123@konkuk.ac.kr

**Supplementary data**

**
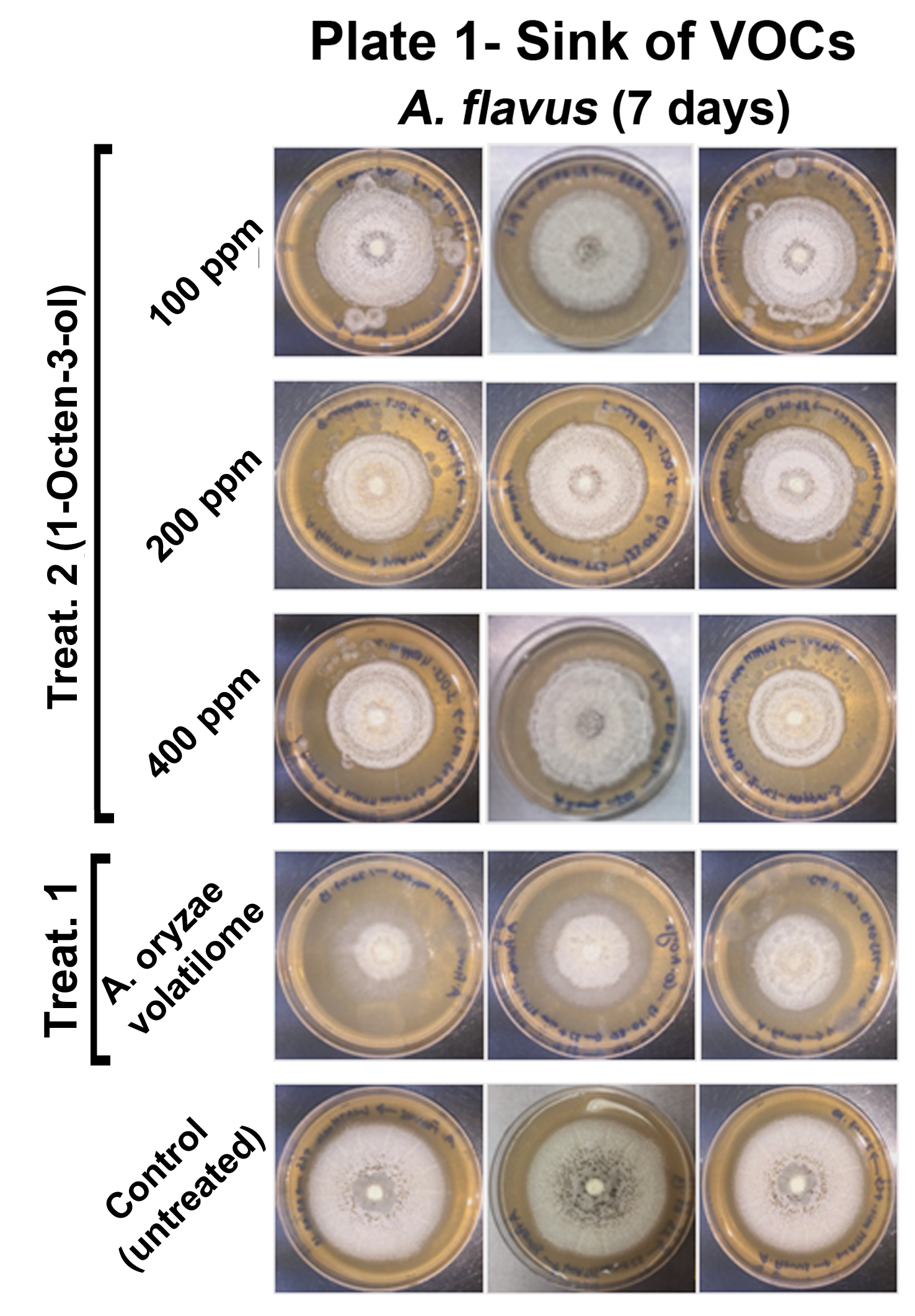
**

**Supplementary Fig. 1.** The growth morphologies of *A. flavus* KCCM 11899 subjected to varying VOC treatments (T1 - *A. oryzae* KCCM 60345 volatilome; T2 - 1-Octen-3-ol gradient titers) on 7-day of incubation.
